# Standardized and quality-assured predictive PD-L1 testing in the upper gastrointestinal tract

**DOI:** 10.1007/s00432-023-05180-5

**Published:** 2023-10-24

**Authors:** Gustavo B. Baretton, Florian Lordick, The Interdisciplinary Expert Group, T. Gaiser, R. Hofheinz, D. Horst, S. Lorenzen, M. Moehler, C. Röcken, P. Schirmacher, M. Stahl, P. Thuss-Patience, K. Tiemann

**Affiliations:** 1 Institute for Pathology, University Hospital Carl Gustav Carus, TU Dresden, Fetscherstrasse 74, 01307 Dresden, Germany; 2 Department of Medicine II (Oncology, Gastroenterology, Hepatology and Pulmonology) and University Cancer Center Leipzig, University of Leipzig Medical Center, Leipzig, Germany; 3 Institute of Applied Pathology, 67346 Speyer, Germany; 4 University Medicine Mannheim, Mannheim, Germany; 5 Institute of Pathology of the Charité-University Medicine Berlin, Berlin, Germany; 6 Department of Medicine III, Klinikum Rechts der Isar, Munich, Germany; 7 Department of Medicine I, University Medicine Mainz, Mainz, Germany; 8 Institute of Pathology, Christian-Albrechts University, Kiel, Germany; 9 Institute of Pathology, University Hospital Heidelberg, Heidelberg, Germany; 10 Department of Oncology and Palliative Care, Kliniken Essen Mitte, Evangelische Huyssens-Stiftung, Essen-Huttrop, Essen, Germany; 11 Charité Center of Tumor Medicine CC14, Charité Campus Virchow-University Medicine Berlin, Berlin, Germany; 12 Institute of Hematopathology, Hamburg, Germany

**Keywords:** PD-L1 testing, Immune checkpoint inhibitor, Predictive biomarkers, Gastroesophageal junction adenocarcinoma, Gastric adenocarcinoma, Esophageal squamous cell carcinoma

## Abstract

As a result of the high approval dynamics and the growing number of immuno-oncological concepts, the complexity of treatment decisions and control in the area of cancers of the esophagus, gastroesophageal junction and stomach is constantly increasing. Since the treatment indication for PD-1 inhibitors that are currently approved in the European Union is often linked to the expression of PD-L1 (programmed cell death-ligand 1), the evaluation of tissue-based predictive markers by the pathologist is of crucial importance for treatment stratification. Even though the immunohistochemical analysis of the PD-L1 expression status is one of the best studied, therapy-relevant biomarkers for an immuno-oncological treatment, due to the high heterogeneity of carcinomas of the upper gastrointestinal tract, there are challenges in daily clinical diagnostic work with regard to implementation, standardization and interpretation of testing. An interdisciplinary group of experts from Germany has taken a position on relevant questions from daily pathological and clinical practice, which concern the starting material, quality-assured testing and the interpretation of pathological findings, and has developed recommendations for structured reporting.

## Introduction

With more than 1.5 million new cases worldwide, esophageal and gastric carcinomas are among the most common cancers (Arnold et al. 2020). The prognosis of advanced or metastatic malignancies in the upper gastrointestinal tract, including carcinomas of the esophagus, the gastroesophageal junction and the stomach, is poor and survival after palliative standard chemotherapy is usually less than 1 year in current clinical studies. In addition, a large proportion of carcinomas are often only diagnosed at an advanced, or metastatic and, therefore, often inoperable stage, and patients can often no longer be treated with curative intention at this point.

The use of immune checkpoint inhibitors (ICI) has expanded the therapeutic options beyond combination chemotherapy alone. Current phase III studies have shown the potential of adding ICI to first-line therapy of locally advanced or metastatic carcinoma in the upper gastrointestinal tract. The recommendation for the use of ICIs is implemented in the current international guidelines (Lordick et al. 2022a; Obermannová et al. 2022; Shah et al. 2023).

In the phase III study KEYNOTE-590, patients with expression of PD-L1 (CPS ≥ 10; combined positive score) showed a significant advantage in overall survival in both advanced esophageal squamous cell carcinoma and HER2 (Human Epidermal Growth Factor Receptor-2)-negative adenocarcinoma of the esophagus and gastroesophageal junction when first-line therapy with pembrolizumab was combined with cisplatin and 5-fluorouracil (5-FU) vs. chemotherapy alone (13.9 vs. 8.8 months; hazard ratio [HR]: 0.57; *p* < 0.0001) (Sun et al. 2021).

In the three-armed CheckMate 648 study, which represented also a phase III study, the first-line therapy in unresectable and advanced, recurrent, or metastatic PD-L1-positive squamous cell carcinoma of the esophagus (TPS ≥ 1%; tumor proportion score) resulted, both for therapy with nivolumab in combination with cisplatin and 5-FU (15.4 vs. 9.1 months; HR: 0.54; *p* < 0.001), as well as for the ICI combination of nivolumab plus ipilimumab alone (13.7 vs. 9.1 months; HR: 0.64; *p* = 0.001), in a significant advantage in overall survival compared to chemotherapy alone (Doki et al. 2022). In another first-line therapy study (CheckMate-649), a significant advantage of combined immune chemotherapy vs. chemotherapy alone (FOLFOX or CapeOx) in the primary endpoint overall survival was demonstrated in HER2-negative adenocarcinoma of the stomach, the gastroesophageal junction or the esophagus: in PD-L1-positive tumors (CPS ≥ 5), the addition of nivolumab led to a median prolongation of survival to 14.4 vs. 11.1 months (HR 0.71; *p* < 0.0001) (Janjigian et al. 2021).

Both the immune checkpoint receptor “programmed cell death-1” (PD-1) and its ligand PD-L1 (programmed cell death-ligand 1) are involved in the regulation of the T-cell response. While the interaction between PD-1 and PD-L1 is essential for maintaining homeostasis and avoiding autoimmunity in the context of a physiological immune response, the above-mentioned interaction taking place in the tumor microenvironment serves as a so-called “immune escape pathway”. An upregulated PD-1 and PD-L1 expression suppresses an active antitumor immune response. Consequently, immune checkpoint inhibition, which inhibits an interaction of PD-1 and PD-L1, and thus enables reactivation of the adaptive immune response, can lead to a competent response of the immune system against the tumor cells and contribute to an improved response to treatment (Mukherji et al. 2022). The expression of PD-L1 on tumor and/or immune cells is one of the best established predictive markers for ICI treatment response. An immunohistochemical determination of PD-L1 expression in tumor biopsies was also carried out in most of the immuno-oncological therapy studies on carcinomas of the esophagus, gastroesophageal junction or stomach (Mukherji et al. 2022; Schoemig-Markiefka et al. 2021).

However, the PD-L1 expression not only differs between various tumor entities, but is also subject to a non-negligible intratumoral heterogeneity; it is also influenced by various biological and methodological factors. At the same time, the immunohistochemical determination of PD-L1 expression offers a nationally available, technically established and economically feasible approach to identify patients who are more likely to benefit from ICI treatment.

With the aim of establishing quality-assured and standardized biomarker diagnostics for the optimal care of tumor patients, a German consortium of experts has developed consensus-based recommendations on the basis of the available literature and many years of practical experience on the following topics:

- predictive biomarkers in the upper gastrointestinal tract
- requirements related to the tumor samples
- dealing with discordant findings
- quality-assured PD-L1 testing

## Predictive biomarkers in the upper gastrointestinal tract

Substantial progress has been made in the understanding of the molecular pathogenesis of carcinomas of the gastrointestinal tract, particularly in the past 10 years, which has subsequently made changes in therapy regimens possible—away from standard chemotherapy based on conventional histomorphological criteria toward targeted therapy additionally controlled by molecular biomarkers (Mukherji et al. 2022; Smith et al. 2021). A major challenge for biomarker-based testing consists, among other factors, in the inter- and intratumoral heterogeneity of the carcinomas (Alsina et al. 2017).

A subtyping proposed by the TCGA consortium (TCGA, The Cancer Genome Atlas) (Epstein–Barr virus-associated [EBV], microsatellite unstable [MSI], genomically stable [GS] or chromosomally unstable [CIN] subtypes) also reflects the molecular heterogeneity of gastric carcinoma (Alsina et al. 2017; Cancer Genome Atlas Research Network 2014), but is currently of minor importance for individualized therapy decisions (Lordick et al. 2022b). In contrast, immunohistological determination for the HER2 expression or the detection of a HER2 gene amplification by means of chromogenic or fluorescence in situ hybridization (CISH/FISH) are already among the established biomarkers in advanced/metastatic gastric cancer (Lordick et al. 2022a; Nagtegaal et al. 2020; Stahl et al. 2022) and adenocarcinomas of the esophagus and gastroesophageal junction (Obermannová et al. 2022; Quezada-Marín et al. 2010). The reported frequency of HER2 overexpression in gastric carcinomas varies between 4.4 and 53.4% (weighted mean: 17.9%) (Jørgensen and Hersom 2012), and in carcinomas of the gastroesophageal junction and esophageal adenocarcinomas between 5 and 30%. This is, amongst other factors, due to heterogeneous intratumoral expression patterns, but also to tumor localization or different HER2 scoring requirements (Pye et al. 2018).

In addition to determination of the HER2 status, clinical practice guidelines currently also recommend evaluating the PD-L1 status—mostly using the CPS (number of positive tumor cells and immune cells) in the case of metastatic gastric carcinoma and/or esophageal carcinoma, also using the TPS, which describes the percentage of PD-L1-expressing tumor cells in relation to all tumor cells found on the respective tumor specimen (Lordick et al. 2022a; Obermannová et al. 2022; Nagtegaal et al. 2020).

Other characteristics, such as MSI or EBV positivity, which are also part of the molecular subtyping of gastric cancer, may be of additional importance in predicting a response to ICI therapy: According to a meta-analysis of four randomized studies, in which the role of MSI on the response to ICI therapy in advanced gastric cancer was evaluated, patients with MSI high (H) status appear to be particularly sensitive to immunotherapy (Pietrantonio et al. 2021). A latent EBV infection, which is assumed to occur in almost 9% of gastric adenocarcinomas, is also discussed as a predictive marker for the response to therapy with ICI (HögnerA 2022). In addition, various markers can be listed, especially in gastric carcinoma, which are potentially relevant to the prognosis, but most of which have not yet found their way into routine application, or the current guidelines: e.g. overexpression of EGFR, c-MET, EGF/TGF-ɑ, VEGF-A or CD44 aberrant transcripts, NTRK gene fusion, reduced expression of E-cadherin or the expression of certain matrix metalloproteinases such as MMP1, MMP7, MMP10. The selection of biomarkers in the area of the upper gastrointestinal tract that are already established for clinical routine is therefore currently still relatively limited (Quezada-Marín et al. 2010).

However, there may be different recommendations and protocols in place that are specific to the individual treatment center, which individually provide for the determination of further molecular markers, such as the EBV status or the tumor mutational burden (TMB) (Mukherji et al. 2022; Halske 2020).

### Consensus

The tissue-based determination of predictive (molecular) biomarkers is subject to an enormous increase in importance and represents an indispensable prerequisite for ICI therapy and the ICI-based therapy concepts that continue to develop with a very high dynamic. It thus makes a central contribution to the resulting significant survival benefits of patients.

**Fig. 1:**
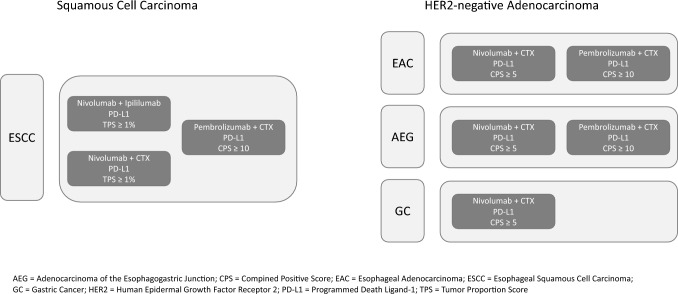
Overview of PD-L1-dependent approvals in first-line locally advanced unresectable or metastatic upper gastrointestinal carcinoma (status of information: April 2023)

However, so-called “reflex testing”, which is independent of the tumor stage, is not recommended for any initial diagnosis, especially in early tumor stages, as long as curative therapy approaches can still be considered: Rather, the predictive biomarker testing should currently be carried out as needed depending on the tumor stage, the immediate therapeutic relevance and the approval status of the available therapeutic approaches, especially since the current therapeutic landscape, including the approval situation, is subject to highly dynamic change (Fig. 1).

Validated predictive biomarkers in adenocarcinoma include

- the immunohistochemical determination of the expression of PD-L1 (CPS and TPS),
- the determination of HER2 expression (and/or HER2 amplification),
- determining the MSI status or mismatch repair status (MMR) in advanced esophageal adenocarcinoma (where MSI is very rare), the gastroesophageal junction adenocarcinoma and adenocarcinoma of the stomach (Lordick et al. 2022a).

Validated as a predictive biomarker in metastatic or locally advanced esophageal squamous cell carcinoma that cannot be curatively treated

- is the immunohistochemical determination of PD-L1 (TPS/CPS) (Obermannová et al. 2022).

## Requirements for the tumor sample material

A high intratumoral heterogeneity or diversity of the tumor cell clones also results in a heterogeneous distribution pattern of the molecular biomarkers to be analyzed in gastroesophageal tumors. Both for esophagogastric adenocarcinomas (Zhou et al. 2020) and for squamous cell carcinomas of the esophagus (Yan et al. 2019), there are signs of considerable intratumoral heterogeneity with regard to PD-L1 expression, with differences between the primary tumor and any lymph node and distant metastases (HögnerA 2022; Yan et al. 2019). In the palliative context, tumor diagnostics and biomarker testing often have to be carried out on limited biopsy material due to the amount of samples available. This tissue material, which often measures only a few millimeters in size, represents only a section of the total tumor mass and harbors the risk of a sampling error.

The assessment of 465 resected tumor tissues from treatment-naïve patients with carcinomas of the stomach or gastroesophageal junction at the Kiel University Department of Pathology (Germany) showed that in 33.1% of cases PD-L1-positive tumor cells were not identified by superficial biopsies of the primary tumor (< 2.5 mm away from the tumor surface), but were only detectable in the tumor center or near the invasion front (Böger et al. 2016). An Asian study identified the minimum number of five central tumor biopsies (with a ≥ 1% cut-off for PD-L1 positivity) required to achieve the highest possible concordance of results obtained by the assessment of biopsies and larger sections of resected tumor tissue. According to the authors, with fewer than five biopsies, sufficient sensitivity and consistency could not be achieved. However, if PD-L1 expression in the unresectable tumor stage can only be determined in biopsies, more than five biopsies may be required (Ye et al. 2020). The updated practice guidelines of the ESMO recommend “multiple endoscopic biopsies”, i.e., specifically five to eight biopsies for gastric carcinoma or ≥ 6 biopsies for esophageal carcinoma, so that sufficient starting material is available for the histological and molecular analysis (Lordick et al. 2022a; Obermannová et al. 2022). In the German S3 guideline for the diagnosis and treatment of gastric cancer, taking at least eight biopsies from all suspected areas is generally recommended if gastric cancer is suspected, whereby the absolute number is less important than the number of tumor-bearing biopsies (Moehler et al. 2019). Since PD-L1 is a dynamic biomarker whose expression can change over the course of the cancer disease and depending on the therapies carried out, the choice of tumor sample in the temporal context of the cancer disease is also crucial (Kraak et al. 2016; Yang et al. 2019; Gao et al. 2017).

In another patient cohort from Kiel/Germany, altered PD-L1 expression under neoadjuvant chemotherapy was observed in neoadjuvantly treated carcinomas of the stomach and gastroesophageal junction (*n* = 141). With overall lower PD-L1 expression, patients with a poor response to neoadjuvant chemotherapy in particular showed an increased expression of PD-L1 compared to the treatment-naïve cohort (Böger et al. 2016) (as well as PD-1 and VISTA [V-domain Ig suppressor of T-cell activation]) (Schoop et al. 2020). Against this background, it makes sense to carry out PD-L1 testing sequentially, especially in the case of an initially negative PD-L1 status, according to the course of the tumor disease over time and especially in case of progression. The material then used for the assessment should be taken from the resected tumor specimen or rebiopsies (also from a metastasis) (Böger et al. 2016). The only meta-analysis to date that has evaluated the conversion of a biomarker status between primary tumors and paired metastases included 38 studies across different tumor entities with regard to PD-L1 conversion. A pooled discordance rate of 22% was determined. Conversions from positive to negative PD-L1 expression status were observed more frequently (41%) than vice versa (16%) (Zou et al. 2021). Discordant findings between the primary tumor and the corresponding metastasis or between biopsy and resected tumor tissue (Kraak et al. 2016), are also known for the HER2 expression status (discordance rate of 9–16% for HER2) (Gumusay et al. 2015).

In principle, there is a risk of sampling error for characteristics that show intratumoral heterogeneity and for biomarkers that are analyzed on biopsy specimens. There is an increased risk of a non-representative, especially false-negative test result in one third of PD-L1-positive gastric carcinomas (Böger et al. 2016). In order to achieve a reduction in this risk, the German expert consortium recommends

- to achieve a representative tissue section with tumor-associated stroma in the resection material,
- if possible, the retrieval of at least five (ideally six to eight) tumor-bearing endoscopic biopsies for the biomarker diagnostics currently required for carcinomas of the upper gastrointestinal tract,
- to take biopsies from different, randomly selected areas of the accessible tumor material.

The endoscopically obtained biopsy is used in the advanced stage of the tumor disease and thus in a frequently palliative therapy situation for the primary diagnosis, and in this situation often represents the only available tissue on which the determination of the biomarkers is carried out. In individual cases, however, the biomarker status should be re-evaluated during the course of the disease and if further material is available, for example from tissue of the resected tumor or metastasis, since the expression of PD-L1 and HER2 changes both during the course of the tumor disease and as a result of the therapies that have been carried out.

Reliable recommendations as to when a rebiopsy or biomarker retest should be carried out cannot currently be formulated uniformly on the basis of the available evidence and require further studies. Due to the high relevance for the therapy decision, however, according to the consistent experiences of the German expert panel, it currently makes sense to repeat biomarker testing

- in biomarker-negative prior biopsy and cases that have been pretreated with chemotherapy and neoadjuvant therapy on a rebiopsy, and to use the biologically latest tumor material or, if it is not accessible, the most up-to-date archive material available
- in case of recurrence on a rebiopsy if the tumor was previously treated or if the previous test result was negative for the primary tumor

According to the current data situation, the therapy decision cannot be made based on biomarker assessment alone, but requires an interdisciplinary overview and classification of all available information and findings in patients.

## Comments on how to deal with discordant findings

In addition to its dynamics, PD-L1 expression is also characterized by a high intratumoral heterogeneity, which entails the risk of sampling errors, especially in biopsy material. This is made even more difficult by the fact that a positive PD-L1 status does not necessarily predict a good response to tumor therapy. PD-L1-negative tumors (including possibly false-negative findings due to tumor heterogeneity, or cases with discordant expression patterns between the primary tumor and metastasis) may respond to ICI-based treatment, or tumors that test positive may not respond. In addition, in clinical studies by various manufacturers in the different tumor entities, their own scoring algorithms and cut-off values were determined and established, which can also make reliable comparability more difficult.

It should be noted that the certainty of results increases with a growing number of tumor-bearing biopsies available and that the risk of sampling errors and thus false-negative results decreases, which is also reflected in the recommendation for the number of samples to be analyzed (Schoemig-Markiefka et al. 2021; Ye et al. 2020).

### Consensus

The treatment decision and classification of the results of the biomarker testing is subject to the treating clinician. Especially in the case of progression of the disease, different results of the biomarker testing in the course, or previous therapy, an individual assessment is required from the clinician side to determine the indication for a (re)biopsy or a retest. The pathological findings provide valuable information for the relevant therapy decision, so that, according to the German expert panel, the following information should be documented and transmitted in a structured manner:

- evaluated material (current material/archive material, resected tumor/(re)biopsy, primary tumor/metastasis),
- information about the available number of tumor-bearing samples,
- if applicable, to point out factors that limit the representativeness of the pathological finding,
- the primary antibody (clone) used and the platform or slide stainer used for the traceability of colleagues,
- scoring and results as absolute and specific numerical values (CPS and TPS) regardless of the tumor entity and, if necessary, information on discordant findings.

In order to make the diagnosis as reliable as possible, the clinician should also provide information on the patient’s history (especially on previous therapy) and therapy planning (e.g., “ICI therapy is planned”).

## Instructions for quality-assured PD-L1 testing

A variety of diagnostic antibodies and assays for PD-L1 immunohistochemistry are available on the market. Even if clinical studies have been carried out with certain antibody clones or assays and kits selected depending on the manufacturer and the tumor entity, there is no compelling need in Germany to also select these antibodies/assays/kits in everyday clinical practice: pathologists in Germany are free to select their diagnostic/medical method and therefore free choice of the test system. The situation in Germany is, therefore, fundamentally different from the testing situation in the USA, for example, where the FDA-defined “companion diagnostics” stipulate the use of certain antibody tests in connection with a clinical question and the approval of a drug. A basic comparability and, above all, reproducibility of the results for the antibodies and platforms used and validated in the various clinical studies is given, which is also supported by the results of one of the first published harmonization studies in gastric carcinoma (22C3, SP263) (Dabbagh and Sughayer 2021). Due to the complexity of PD-L1 testing, the interpretation of the immunohistochemical PD-L1 examinations requires in-depth knowledge and appropriate training for the corresponding scores and cut-offs of the different tumor entities (Deutsche Akkreditierungsstelle (DAkkS) 2015). The establishment of a standardized, reproducible immunohistochemistry is, therefore, of paramount importance, which can be achieved, among other factors, by regularly participating in external ring tests (e.g. as provided by the quality assurance initiative “Pathologie QuIP GmbH” in Germany) and accreditation as well as quality assurance measures not related to round robin tests (e.g., NordicQC) contributing to maintaining quality and improvement. A comprehensive quality assurance of molecular pathological diagnostics is part of high-quality clinical cancer care (Wenzel et al. 2021). Participation in appropriate training courses for pathologists represents an additional cornerstone to ensure a high quality of results.

### Consensus

When selecting the test systems for PD-L1 immunohistochemistry, German pathologists are free to choose between the methods. For pre-analytical processing, reference can be made to the specifications, which are available in this respect from the test provider. Training of the pathologists involved in PD-L1 evaluation and regular participation in external round robin tests on PD-L1 immunohistochemistry is important. So far, there are no common or binding specifications for the processing time from the receipt of specimen to the finished and transmitted report of the pathological findings, although despite the complexity of pathological diagnostics described, the report should usually be completed within 3–5 working days.
